# Inter-Relay Interference Mitigation for Chirp-Based Two-Path Successive Relaying Protocol †

**DOI:** 10.3390/s19153346

**Published:** 2019-07-30

**Authors:** Kwang-Yul Kim, Yoan Shin

**Affiliations:** School of Electronic Engineering, Soongsil University, Seoul 06978, Korea

**Keywords:** chirp spread spectrum, two-path successive relaying, inter-relay interference, multiple linear chirp, cross-correlation coefficient

## Abstract

Since the chirp spread spectrum (CSS) system is considered as a communication technology for the Internet of things (IoT), long-range communication and a high data rate are required. In wireless communications, in order to increase spectral efficiency and to extend transmission coverage, a two-path successive relaying (TPSR) protocol has been proposed. Thus, in order to improve transmission performance of the CSS system, in this paper we apply the TPSR protocol to the CSS system. However, since the TPSR protocol is successively relaying data, the spectral efficiency may be limited due to inter-relay interference (IRI). Hence, we propose a multiple linear chirp-based IRI mitigation method for the CSS-based TPSR protocol. In the proposed scheme, the cross-correlation coefficient (CCC) has been derived mathematically according to a separating bandwidth in a given total bandwidth. Then, one separating bandwidth that guarantees the transmission performance is allocated to the primary relay by considering a single relay CCC (SR-CCC) and another separating bandwidth that guarantees the orthogonality from the primary relay is allocated to the secondary relay by considering the inter-relay CCC (IR-CCC). Since the IR-CCC means a degree of similarity between these two relays, it is possible to mitigate the IRI effect within the same bandwidth by allocating orthogonal separating bandwidths to each relay. Simulation results show that the proposed scheme can improve the transmission performance by mitigating the IRI effect even in high IRI environments. Consequently, we expect that the proposed scheme can extend the transmission coverage and increase the data rate of the CSS system.

## 1. Introduction

The spread spectrum system was firstly developed to improve the low probability of detection and anti-jamming capability of military wireless communications [1]. Since the spread spectrum system has robustness against multipath fading as well as multiple access, it has been used as a core technology for code division multiple access (CDMA). Among the spread spectrum systems, chirp spread spectrum (CSS) system using a chirp signal has several advantages such as low energy consumption, high resolution, and Doppler effect robustness. Due to these advantages, the CSS system has been used for military radio detection and ranging (RADAR) systems from the 1940s [2] and adopted as an IEEE 802.15.4a standard for a physical layer of wireless localization systems [3]. Recently, it is mainly used for a long range (LoRa) technology of low-power wide area networks for Internet of Things (IoT) communications [4]. The transmission method for the CSS system is generally classified into a direct modulation-based CSS (DM-CSS) scheme and a binary orthogonal keying-based (BOK-CSS) scheme, depending on the usage of the chirp signal [5]. In the DM-CSS scheme, the data modulation part and the chirp generation part are independently separated, and the chirp signal is used as a spreading code similar to a pseudo-noise sequence in direct sequence spread spectrum systems. Although it has the advantage of obtaining antipodal characteristics according to the modulation technique, there is a disadvantage that the complexity is increased to synchronize precisely between the separated parts. On the other hand, the BOK-CSS scheme transmits a single linear chirp (SLC) according to binary data. The SLC mainly uses an up-chirp, which increases the instantaneous frequency or a down-chirp, which decreases the instantaneous frequency. Therefore, the complexity of the BOK-CSS system is relatively low compared to the DM-CSS scheme. However, since the SLC-based BOK-CSS scheme cannot guarantee perfect orthogonality, various studies have been carried out to analyze the cross-correlation coefficient (CCC) to improve transmission performance [6,7,8]. In [6], the authors consider the CCC for multi-user chirp signals. In [7], a study on the CCC of nonlinear chirp is conducted to overcome the limitation of the linear chirp that cannot obtain a negative CCC. In [8], the authors analyzed and derived the optimal CCC by using a multiple linear chirp (MLC). Moreover, in order to improve the data rate, [9] proposed the overlapped chirp transmission scheme. In [9], it is shown that the data rate can be improved by overlapping a plurality chirp with different overlap time.

In wireless communications, in order to obtain diversity gain and to extend transmission coverage, cooperative relay-based communication has been widely studied [10,11]. In the cooperative relay schemes, there are two main issues: relay power allocation and relay selection [12]. In the relay power allocation technique, since the relay power controls not only the transmission coverage but also the transmission performance, various research has been done to optimize the relaying power [13,14]. In the relay selection technique, since a wireless channel environment is randomly fluctuated, an opportunistic relay selection (ORS) protocol that selects the optimal relay among multiple relays based on channel state has been proposed [15]. In order to operate the ORS protocol, there are several objectives for selecting the optimal single relay, such as the minimum bit error rate (BER), the maximum system throughput [16], and the minimum power consumption [17]. Moreover, several relay selection techniques for the ORS protocol, such as max–min relay selection, harmonic mean relay selection, and threshold-based selection, have been studied [18]. The common advantage of these ORS protocols is that they do not need to consider an inter-relay interference (IRI) effect because they select only one relay. However, the disadvantage of the ORS protocol is that the spectral efficiency may be decreased because of half-duplex transmission. Thus, in order to overcome orthogonal relaying time and to increase the spectral efficiency, a two-path successive relaying (TPSR) protocol has been proposed [19]. In the TPSR protocol, when one relay receives data from the transmitter, then another one successively relays data to the receiver. However, since the TPSR protocol can virtually perform full duplex transmission by the successive relaying, it can cause the IRI problem [20]. Therefore, various studies have been carried out to solve the IRI problem for the TPSR protocol [20,21]. In [20], the authors proposed power allocation with only a pair of relays under both individual and global power constraints. Moreover, the relay pair selection is performed with a criterion based on their proposed power allocation. In [21], a cognitive radio approach is proposed to assign a priority to a relay having a relatively better channel state and the relay having the priority is allocated with a maximum relaying power. In this scheme, it is confirmed that the spectral efficiency can be improved over the conventional TPSR scheme even in a high IRI environment by opportunistically assigning the priority according to the channel state.

We described earlier the requirements for the CSS system and the TPSR protocol to improve their performance. The TPSR protocol can be considered as a method to improve the spectral efficiency. In the CSS system, if the system guarantees the optimal CCC between different chirp signals, the interference occurring at the transmitter can be reduced. Thus, in this paper, we consider the CSS-based TPSR protocol and propose a method to mitigate the IRI effect. The proposed scheme considers the MLC that can adjust the CCC according to the separating bandwidth to solve the IRI problem naturally occurring in the TPSR protocol. In [22], we proposed the concept of the CSS-based TPSR protocol and analyzed the BER performance of the primary relay. However, in [22] we did not derive the IRI effect theoretically and did not analyze the BER performance of the secondary relay. Thus, in order to analyze the IRI effect theoretically, we derive the inter-relay CCC (IR-CCC) according to the separating bandwidth when the single relay CCC (SR-CCC) is given. Then, we express the theoretical BER performance of both relays and describe the physical meaning of the SR-CCC and the IR-CCC. The following is a summary of the contributions of this paper.

1. To the best of the authors’ knowledge, no research using the relay node in the CSS system has yet been carried out.
2. When the operator designs the relay-based CSS system, our analysis can easily offer the parameters to mitigate the IRI effect.
3. In the CSS-based TPSR protocol, it is expected that the proposed scheme can increase the data rate and extend the transmission coverage even in a high IRI environment.

The rest of the paper is organized as follows. In Section 2, the BOK-CSS scheme and the TPSR protocol model are described. Then, the IR-CCC according to the separating bandwidth and the IRI analysis are described in Section 3. In Section 4, the simulation results are presented, followed by the conclusion in Section 5.

## 2. System Model

### 2.1. BOK-CSS Scheme

**Figure 1:**
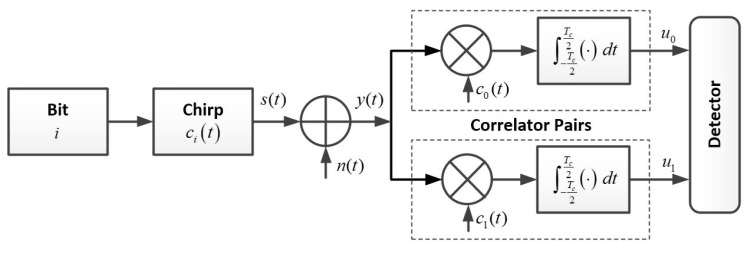
Conventional binary orthogonal keying-based chirp spread spectrum (BOK-CSS) scheme.

Figure 1 shows the conventional BOK-CSS scheme in the additive white Gaussian noise (AWGN) channel [5]. The transmitted signal s(t) is represented as one of two chirp signals according to the input bit i∈{0,1}. The SLC $c_{i}\left(t\right)$ for the bit *i* used in the BOK-CSS scheme can be expressed as [5,7]

(1) $$\begin{array}{c} c_{i}\left(t\right)=\cos\left(2\pi f_{c}t-{\left(-1\right)}^{i}\pi \mu t^{2}\right),\left|t\right|⩽\frac{T_{c}}{2}, \end{array}$$

where $T_{c}$ denotes the chirp duration in sec, $f_{c}$ denotes the center frequency in Hz, and μ denotes the chirp rate in Hz/sec, which is the same as the frequency sweep rate. Then, if the chirp rate takes a positive direction by μ>0 (or a negative direction by μ<0), the transmitted signal represents the up-chirp (or the down-chirp) and the instantaneous frequency increases (or decreases). We consider the AWGN channel with the noise n(t). At the receiver, when the received signal y(t)=s(t)+n(t) is passed through the correlator pairs, the outputs of the correlators are obtained:

(2) $$\begin{array}{c} u_{i}=\int_{-\frac{T_{c}}{2}}^{\frac{T_{c}}{2}}c_{i}\left(t\right)\cdot y\left(t\right)\;dt,\;i\in \left\{0,1\right\}. \end{array}$$

Since the detector decides the transmitted bit by comparing the bit energy, the energy of the difference signal should be analyzed. The energy of the difference signal is [23]

(3) $$\begin{array}{c} E_{d}=\int_{-\frac{T_{c}}{2}}^{\frac{T_{c}}{2}}\left[c_{1}\left(t\right)-c_{0}\left(t\right)\right]dt=\int_{-\frac{T_{c}}{2}}^{\frac{T_{c}}{2}}c_{1}^{2}\left(t\right)\;dt+\int_{-\frac{T_{c}}{2}}^{\frac{T_{c}}{2}}c_{0}^{2}\left(t\right)\;dt+\int_{-\frac{T_{c}}{2}}^{\frac{T_{c}}{2}}c_{1}\left(t\right)\cdot c_{0}\left(t\right)\;dt. \end{array}$$

Here, since each of the first two terms denotes the bit energy $E_{b}$, (3) is simplified to

(4) $$\begin{array}{c} E_{d}=2E_{b}-2\rho E_{b}=2E_{b}\left(1-\rho \right), \end{array}$$

where ρ is the SR-CCC of the SLC when considering a single relay environment, which is given by

(5) $$\begin{array}{c} \rho =\frac{1}{E_{b}}\int_{-\frac{T_{c}}{2}}^{\frac{T_{c}}{2}}c_{1}\left(t\right)\cdot c_{0}\left(t\right)\;dt. \end{array}$$

In order to analyze the SR-CCC of the SLC, various values of the time-bandwidth product $T_{c}B$ are generally considered. The SR-CCC of the SLC according to $T_{c}B$ can be expressed as [24]

(6) $$\begin{array}{c} \rho =\frac{1}{\sqrt{T_{c}B}}C\left(\sqrt{T_{c}B}\right), \end{array}$$

where $C\left(x\right)=\int_{0}^{x}\cos\left(\frac{\pi v^{2}}{2}\right)dv$ is the Fresnel’s cosine integral [5]. Consequently, the theoretical BER of the SLC is affected by the SR-CCC and is finally obtained as follows [23].

(7) $$\begin{array}{c} P_{e}=Q\left(\sqrt{\frac{E_{d}}{2N_{0}}}\right)=Q\left(\sqrt{\frac{E_{b}\left(1-\rho \right)}{N_{0}}}\right), \end{array}$$

where $Q\left(x\right)≜\frac{1}{\sqrt{2\pi }}\int_{x}^{\infty }e^{-t^{2}/2}dt$. Note that since the SR-CCC of the SLC cannot obtain the orthogonality $\left(\rho =0\right)$, the antipodal $\left(\rho =-1\right)$ case is the best for a single relay environment.

### 2.2. Two-Path Successive Relaying Protocol

**Figure 2:**
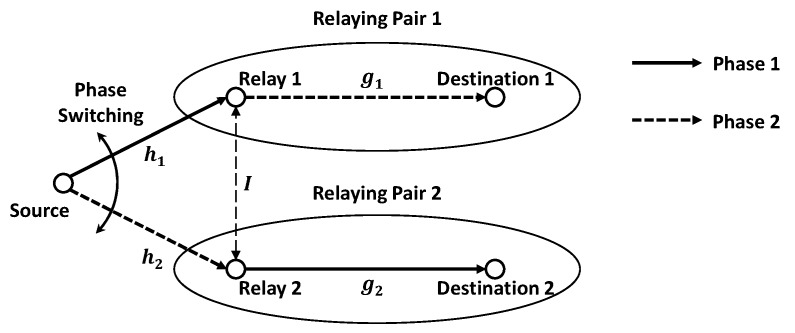
Conventional two-path successive relaying (TPSR) protocol model.

Figure 2 shows the conventional TPSR protocol model [21]. It consists of a single source, two amplify-and-forward (AF) relays, and two destinations. We assume that a relay and a destination are determined as a relaying pair in advance through the scheduling process, and the source alternatively transmits the data to each relay. Note that although two relaying pairs are generally considered in the single TPSR protocol environment, it is possible to extend this protocol to a more complex scenario such as multiple TPSR protocol environments by considering the relay selection process such as in [25]. In the first phase, relay 2 relays the data to its destination 2 while the source transmits the data to relay 1. In the second phase, relay 1 relays the data to its destination 1 while the source transmits the data to relay 2. In Figure 2, $h_{k}$ $\left(k\in \left\{1,2\right\}\right)$ denotes the channel gain between the source and the *k*-th relay, $g_{k}$ denotes the channel gain between the *k*-th relaying pair, and *I* denotes the channel gain of the IRI. We assume that there is no direct path between the source and each destination. In this paper, since the relay receives the data from the source and another relay at the same time, we handled the IRI problem as being the same as a co-channel interference problem. Thus, if we consider the AWGN channel, the theoretical BER of the TPSR protocol can be calculated as [26]

(8) $$\begin{array}{cc} P_{\mathrm{IRI}} & =\frac{1}{2}Q\left(\sqrt{\gamma }+\alpha \sqrt{\gamma }\right)+\frac{1}{2}Q\left(\sqrt{\gamma }-\alpha \sqrt{\gamma }\right)\\ & =\frac{1}{2}Q\left(\left(1+\alpha \right)\sqrt{\gamma }\right)+\frac{1}{2}Q\left(\left(1-\alpha \right)\sqrt{\gamma }\right), \end{array}$$

where γ=S/N is the signal-to-noise ratio (SNR), *S* is the average power of the received signal, *N* is the average power of the noise, $\alpha =1/\sqrt{S/I}$ is the magnitude of the IRI, and $S/I=1/\alpha^{2}$ is the average signal-to-interference ratio. Here, the SNR is $\gamma =\left(E_{b}\left(1-\rho \right)/N_{0}\right)\cdot \left(B/R_{b}\right)$, $N_{0}$ is the noise power spectral density in W/Hz, $B/R_{b}$ is the processing gain $G_{p}$, *B* is the transmission (or spreading) bandwidth, and $R_{b}$ is the bit rate. From (8), since the SLC cannot guarantee the orthogonality not only in the SR-CCC but also in the IR-CCC, the BER may be significantly deteriorated when the IRI effect is increased. On the other hand, since the MLC can guarantee the orthogonality in both the SR-CCC and IR-CCC, the BER performance can be significantly improved because the IRI effect can be directly reduced. Moreover, since each relay successively transmits the data, the IR-CCC should be considered more than the SR-CCC in the TPSR protocol.

## 3. MLC Signal Model and IRI Analysis

In order to mitigate the IRI effect, we consider the MLC, which can simply adjust the CCC, and analyze the IRI effect by deriving the IR-CCC according to the separating bandwidth.

### 3.1. MLC Signal Model

In order to successively transmit and receive the data in the TPSR protocol, we consider the MLC-based BOK-CSS scheme in which the source and two relays transmit the data with a different separating bandwidth. The MLC for the *k*-th relay according to the bit *i* is [27]

(9) $$c_{f_{i,k}}\left(t_{1}\right)=A\cdot \cos\left(2\pi f_{0}t_{1}+\pi \mu_{f_{i,k}}t_{1}^{2}\right),$$

(10) $$c_{b_{i,k}}\left(t_{2}\right)=A\cdot \cos\left(2\pi \left(f_{0}+B_{f_{i,k}}\right)\left(t_{2}-\frac{T_{c}}{2}\right)+\pi \mu_{b_{i,k}}{\left(t_{2}-\frac{T_{c}}{2}\right)}^{2}\right),$$

where $A=\sqrt{E_{s}/T_{c}}$, $E_{s}$ is the chirp energy, and $0⩽t_{1}⩽T_{c}/2$ and $T_{c}/2⩽t_{2}⩽T_{c}$ are the duration of the front-chirp $c_{f_{i,k}}\left(t_{1}\right)$ and the back-chirp $c_{b_{i,k}}\left(t_{2}\right)$, respectively. Moreover, $f_{0}$ is the initial frequency, $B_{f_{i,k}}=B/2+{\left(-1\right)}^{i}\left\{B_{\ell }/2+\left(k-1\right)M_{\ell }/2\right\}$ is the allocated separating bandwidth for the *k*-th relay according to the bit *i*, $B_{\ell }=\left|B_{f_{1,1}}-B_{f_{0,1}}\right|$ is the primary separating bandwidth between the chirps of the primary relay, and $M_{\ell }=\left|B_{f_{1,2}}-B_{f_{0,2}}\right|$ is the secondary separating bandwidth between the chirps of the secondary relay. Then, the front-chirp rate $\mu_{f_{i,k}}$ and the back-chirp rate $\mu_{b_{i,k}}$ are respectively obtained as follows.

(11) $$\mu_{f_{i,k}}=B_{f_{i,k}}/\left(T_{c}/2\right),$$

(12) $$\mu_{b_{i,k}}=\left(B-B_{f_{i,k}}\right)/\left(T_{c}/2\right).$$

### 3.2. IRI Analysis

#### 3.2.1. Derivation of IRI

In order to improve the BER performance of the BOK-CSS scheme in a single user environment, we derived the SR-CCC of the MLC according to the separating bandwidth [24]. However, in order to mitigate the IRI effect occurring in the TPSR protocol, we propose the IR-CCC concept, which considers the similarity of the MLC between two relays. Thus, in order to analyze the IRI effect, we need to analytically derive the IR-CCC for both cases where two relays transmit the same bits and the different bits. The IR-CCC when two relays transmit the same bits is

(13) $$\begin{array}{cc} \rho^{s} & =\rho_{f}^{s}+\rho_{b}^{s}\\ & =\frac{1}{\frac{E_{s}}{2}}\int_{0}^{\frac{T_{c}}{2}}c_{f_{1,1}}\left(t\right)\cdot c_{f_{1,2}}\left(t\right)dt+\frac{1}{\frac{E_{s}}{2}}\int_{\frac{T_{c}}{2}}^{T_{c}}c_{b_{1,1}}\left(t\right)\cdot c_{b_{1,2}}\left(t\right)dt. \end{array}$$

Here, we use the relationship of $\cos\left(a\right)\cos\left(b\right)=1/2\left(\cos(a-b)+\cos(a+b)\right)$, then (13) becomes

(14) $$\rho_{\ell }^{s}=\frac{1}{T_{c}}\int_{0}^{\frac{T_{c}}{2}}\cos\left(\pi \mu_{\ell }^{s}t^{2}\right)dt+\frac{1}{T_{c}}\int_{\frac{T_{c}}{2}}^{T_{c}}\cos\left(\pi \left(\mu_{\ell }^{s}t^{2}+2B_{\ell }^{s}t\right)\right)dt,$$

where $B_{\ell }^{s}=\left|B_{f_{1,1}}-M_{\ell }^{s}\right|$, $M_{\ell }^{s}=\left\{B/2,B/2+D_{\Delta },B/2+2D_{\Delta },\cdots ,B\right\}$ is a set of the secondary separating bandwidths for the secondary relay when i=1, $\mu_{\ell }^{s}=B_{\ell }^{s}/\left(T_{c}/2\right)$ is the difference of the chirp rate between the primary relay and the secondary relay when they transmit the same bits, and $D_{\Delta }$ is the bandwidth separation interval.

Similarly, the IR-CCC when two relays transmit the different bits is

(15) $$\begin{array}{cc} \rho^{d} & =\rho_{f}^{d}+\rho_{b}^{d}\\ & =\frac{1}{\frac{E_{s}}{2}}\int_{0}^{\frac{T_{c}}{2}}c_{f_{1,1}}\left(t\right)\cdot c_{f_{0,2}}\left(t\right)dt+\frac{1}{\frac{E_{s}}{2}}\int_{\frac{T_{c}}{2}}^{T_{c}}c_{b_{1,1}}\left(t\right)\cdot c_{b_{0,2}}\left(t\right)dt. \end{array}$$

Here, we also use the relationship of the triangular functions. Then (15) can be expressed as

(16) $$\rho_{\ell }^{d}=\frac{1}{T_{c}}\int_{0}^{\frac{T_{c}}{2}}\cos\left(\pi \mu_{\ell }^{d}t^{2}\right)dt+\frac{1}{T_{c}}\int_{\frac{T_{c}}{2}}^{T_{c}}\cos\left(\pi \left(\mu_{\ell }^{d}t^{2}+2B_{\ell }^{d}t\right)\right)dt,$$

where $B_{\ell }^{d}=\left|B_{f_{1,1}}-M_{\ell }^{d}\right|$, $M_{\ell }^{d}=\left\{B/2,B/2-D_{\Delta },B/2-2D_{\Delta },\cdots ,0\right\}$ is a set of the secondary separating bandwidths for the secondary relay when i=0, and $\mu_{\ell }^{d}=B_{\ell }^{d}/\left(T_{c}/2\right)$ is the difference of the chirp rate between the primary relay and the secondary relay when they transmit the different bits.

Since the representations of the IR-CCC when transmitting the same bits and different bits are similar, it is convenient to analyze both cases together. At first, in order to derive the IR-CCC of the front-chirp, the first terms in (14) and (16) are expressed together as

(17) $$\rho_{f,\ell }=\frac{1}{T_{c}}\int_{0}^{\frac{T_{c}}{2}}\cos\left(\pi \mu_{\ell }t^{2}\right)dt.$$

With a change of the variable $v=\sqrt{2\mu_{\ell }}t$, (17) is simplified to

(18) $$\rho_{f,\ell }=\frac{1}{T_{c}\sqrt{2\mu_{\ell }}}\int_{0}^{\frac{T_{c}}{2}\sqrt{2\mu_{\ell }}}\cos\left(\pi \frac{v^{2}}{2}\right)dv.$$

Then, with a relationship of the chirp rate $\mu_{\ell }=B_{\ell }/T_{c}$, the IR-CCC of the front-chirp is finally obtained by

(19) $$\rho_{f,\ell }=\frac{1}{2\sqrt{T_{c}B_{\ell }}}C\left(\sqrt{T_{c}B_{\ell }}\right).$$

Next, in order to derive the IR-CCC of the back-chirp, the second terms in (14) and (16) are expressed together as

(20) $$\begin{array}{cc} \rho_{b,\ell } & =\frac{1}{T_{c}}\int_{\frac{T_{c}}{2}}^{T_{c}}\cos\left(\pi \left(\mu_{\ell }t^{2}+2B_{\ell }t\right)\right)dt\\ & =\frac{1}{T_{c}}\int_{\frac{T_{c}}{2}}^{T_{c}}\cos\left(\pi \left({\left(\sqrt{\mu_{\ell }}t+\frac{B_{\ell }}{\sqrt{\mu_{\ell }}}\right)}^{2}-\frac{B_{\ell }^{2}}{\mu_{\ell }}\right)\right)dt. \end{array}$$

Here, we use the relationship of cos(a−b)=cos(a)cos(b)+sin(a)sin(b), then (20) becomes

(21) $$\rho_{b,\ell }=\frac{1}{T_{c}}\int_{\frac{T_{c}}{2}}^{T_{c}}\cos\left(\pi {\left(\sqrt{\mu_{\ell }}t+\frac{B_{\ell }}{\sqrt{\mu_{\ell }}}\right)}^{2}\right)\cdot \cos\left(\pi \frac{B_{\ell }^{2}}{\mu_{\ell }}\right)+\sin\left(\pi {\left(\sqrt{\mu_{\ell }}t+\frac{B_{\ell }}{\sqrt{\mu_{\ell }}}\right)}^{2}\right)\cdot \sin\left(\pi \frac{B_{\ell }^{2}}{\mu_{\ell }}\right)dt.$$

With a change of the variable $w=\sqrt{2}\left(\sqrt{\mu_{\ell }}t+\frac{B_{\ell }}{\sqrt{\mu_{\ell }}}\right)$ and the chirp rate $\mu_{\ell }=B_{\ell }/T_{c}$, the IR-CCC of the back-chirp is finally obtained by

(22) $$\rho_{b,\ell }=\frac{1}{2\sqrt{T_{c}B_{\ell }}}\left[\cos\left(\pi \frac{T_{c}B_{\ell }}{2}\right)C\left(\sqrt{T_{c}B_{\ell }}\right)+\sin\left(\pi \frac{T_{c}B_{\ell }}{2}\right)S\left(\sqrt{T_{c}B_{\ell }}\right)\right],$$

where $S\left(x\right)=\int_{0}^{x}\sin\left(\frac{\pi v^{2}}{2}\right)dv$ is the Fresnel’s sine integral [5]. Consequently, each IR-CCC for the same bits or difference bits is given by the following.

(23) $$\rho_{\ell }^{s}=\frac{1}{2\sqrt{T_{c}B_{\ell }^{s}}}\left[C\left(\sqrt{T_{c}B_{\ell }^{s}}\right)\cdot \left(1+\cos\left(\pi \frac{T_{c}B_{\ell }^{s}}{2}\right)\right)+\sin\left(\pi \frac{T_{c}B_{\ell }^{s}}{2}\right)S\left(\sqrt{T_{c}B_{\ell }^{s}}\right)\right],$$

(24) $$\rho_{\ell }^{d}=\frac{1}{2\sqrt{T_{c}B_{\ell }^{d}}}\left[C\left(\sqrt{T_{c}B_{\ell }^{d}}\right)\cdot \left(1+\cos\left(\pi \frac{T_{c}B_{\ell }^{d}}{2}\right)\right)+\sin\left(\pi \frac{T_{c}B_{\ell }^{d}}{2}\right)S\left(\sqrt{T_{c}B_{\ell }^{d}}\right)\right].$$

#### 3.2.2. IRI Effect on BER Performance

Since the IR-CCC means the degree of similarity of the MLC between the relays, the IRI magnitude α in (8) is directly influenced by the IR-CCC. Then, (8) can be changed to

(25) $$P_{\mathrm{IRI},\ell }=\frac{1}{2}Q\left(\left(1+\alpha |\beta_{\ell }|\right)\sqrt{\frac{E_{b}\left(1-\rho_{\ell }\right)}{N_{0}}G_{p}}\right)+\frac{1}{2}Q\left(\left(1-\alpha |\beta_{\ell }|\right)\sqrt{\frac{E_{b}\left(1-\rho_{\ell }\right)}{N_{0}}G_{p}}\right),$$

where $\beta_{\ell }$ is the IR-CCC of the MLC, which is the normalized IRI ratio at the *ℓ*-th separating bandwidth expressed as

(26) $$\beta_{\ell }=\frac{\rho_{\ell }^{s}-\rho_{\ell }^{d}}{\max_{\ell \in \left\{1,\cdots ,L\right\}}\left(\rho_{\ell }^{s}-\rho_{\ell }^{d}\right)},$$

and $\rho_{\ell }$ is the SR-CCC of the MLC at the *ℓ*-th separating bandwidth expressed as [24]

(27) $$\begin{array}{c} \rho_{\ell }=\frac{1}{2\sqrt{T_{c}B_{\ell }}}\left[C\left(\sqrt{T_{c}B_{\ell }}\right)\cdot \left(1+\cos\left(\pi \frac{T_{c}B_{\ell }}{2}\right)\right)+\sin\left(\pi \frac{T_{c}B_{\ell }}{2}\right)S\left(\sqrt{T_{c}B_{\ell }}\right)\right]. \end{array}$$

From a signal characteristic perspective, since the SR-CCC of the MLC cannot obtain the antipodal property, it has a limitation to improve the BER compared to the antipodal signal in a single relay environment. On the other hand, in the TPSR protocol, if the system considers the IR-CCC with the orthogonal property $\left(|\beta_{\ell }|\approx 0\right)$, we expect that the IRI effect can be significantly mitigated and the BER performance of the MLC can be the same as the AWGN channel according to the SR-CCC of the MLC. Consequently, the proposed scheme can improve the BER performance over the antipodal-based TPSR protocol in high IRI environments.

## 4. Simulation Results

In order to evaluate the BER performance, we considered the BOK-CSS scheme given in Figure 1 and the TPSR protocol given in Figure 2. For the computer simulations, the chirp duration $T_{c}=1\;\mu s$, the bit rate $R_{b}=1\;\mathrm{Mbps}$, and the spreading bandwidth B=30MHz were considered. The BER performance was measured both for the primary relay and the secondary relay to analyze the IRI effect occurring from each relay, and then the theoretical BER performance was obtained from (25). Since the purpose of our paper is to analyze and mitigate the IRI effect, three kinds of MLC combinations as the representative cases were chosen based on the analyses of the SR-CCC and the IR-CCC. Then, we considered the SLC-based TPSR (“SLC-TPSR”) and the antipodal-based TPSR (“Antipodal-TPSR”) to compare the BER performance to these MLC combinations (“MLC-TPSR”). We used MATLAB for the simulation and the number of Monte Carlo runs was 1,000,000.

### 4.1. SR-CCC and IR-CCC Values

**Figure 3:**
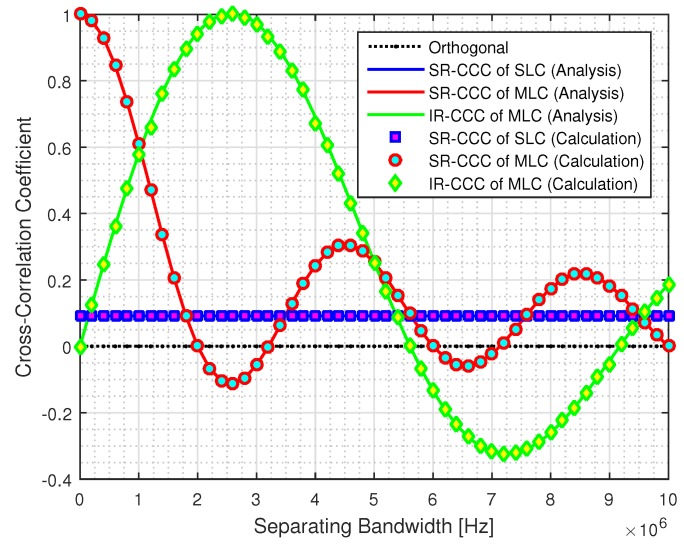
Cross-correlation coefficient (CC) and inter-relay CCC (IR-CCC) values according to separating bandwidth.

Figure 3 shows the SR-CCC and the IR-CCC values according to the separating bandwidth. In Figure 3, since the SLC-TPSR is not affected by the separating bandwidth, the SR-CCC of the SLC is equal to 0.0914 for all separating bandwidths. On the other hand, both the SR-CCC and IR-CCC fluctuated according to the separating bandwidth in the MLC. Moreover, we observe from (25) that the performance of each relay is determined by the SR-CCC when the IRI effect is ignored, and the performance of each relay is determined by the IR-CCC when the IRI effect exists. Since we propose an IRI mitigation method in this paper, we assume the IRI effect and define the separating bandwidth allocation rules for each relay as follows.

- Primary relay: In order to guarantee the best BER performance in a given separating bandwidth, the separating bandwidth with the minimum SR-CCC $\min(\rho_{\ell })$ is preferably allocated to the primary relay.
- Secondary relay
  1. In order to guarantee the BER performance of the secondary relay without considering the IR-CCC, the separating bandwidth at the minimum SR-CCC $\min(\rho_{\ell })$ is allocated to the secondary relay.
  2. In order to consider a negative SR-CCC and negative IR-CCC, the separating bandwidth with these conditions can be allocated to the secondary relay.
  3. In order to guarantee the orthogonality from the primary relay, the separating bandwidth at the IR-CCC $\beta_{\ell }\approx 0$ is allocated to the secondary relay without considering the SR-CCC.

Thus, from the separating bandwidth allocation rules, we observe that the primary separating bandwidth of $B_{\ell }=2.6\;\mathrm{MHz}$ should be occupied to improve the BER performance for the primary relay. Moreover, when the primary relay occupies the primary separating bandwidth of $B_{\ell }=2.6\;\mathrm{MHz}$, the secondary relay can occupy the secondary separating bandwidth of $M_{\ell }=6.6\;\mathrm{MHz}$ to improve the BER performance over the orthogonal counterpart. However, in order to significantly mitigate the IRI effect against the primary relay, the secondary relay should occupy the secondary separating bandwidth of $M_{\ell }=9.2\;\mathrm{MHz}$ that can guarantee the IR-CCC $|\beta_{\ell }|\approx 0$. Table 1 shows the considered parameters of the separating bandwidth pairs for each MLC.

### 4.2. BER Performance with IRI

**Figure 4:**
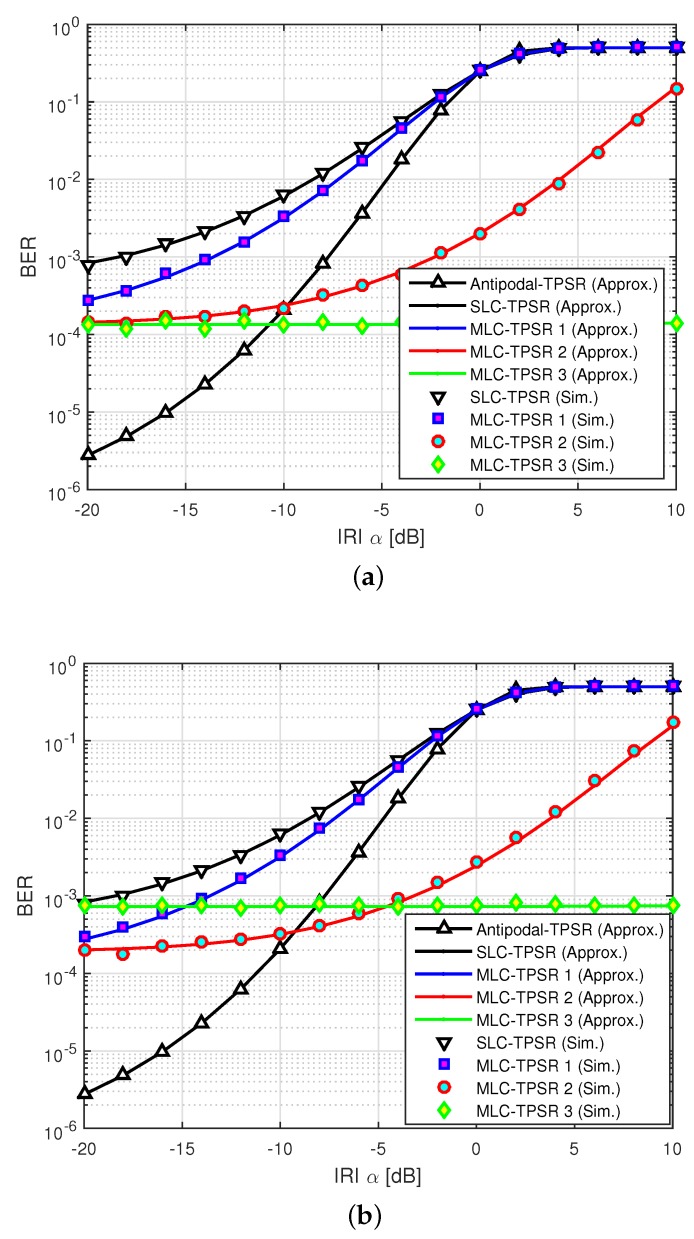
Bit error rate (BER) performance according to inter-relay interference (IRI) $\left(E_{b}/N_{0}=-4\;\mathrm{dB},\;G_{p}=30\right)$. (**a**) Primary relay; (**b**) secondary relay.

Figure 4 shows the BER performance according to the IRI magnitude α when $E_{b}/N_{0}=-4\;\mathrm{dB}$ with $G_{p}=30$. At first, we analyze the BER performance of the primary relay. From the results, it is observed that when each relay uses the SLC-TPSR, the BER performance is significantly degraded due to the IR-CCC of $|\beta_{\ell }|=1$, meaning that the system cannot directly reduce the IRI effect. Moreover, in the case of the MLC-TPSR 1, due to the IR-CCC of $|\beta_{\ell }|=1$, like the SLC-TPSR and the Antipodal-TPSR, the BER performance is significantly degraded even in low IRI environments and is worse than the BER of $10^{-3}$ from around α=−18dB. However, since the Antipodal-TPSR has the antipodal property, the BER performance is improved compared to the SLC-TPSR and the MLC-TPSR 1, and is worse than the BER of $10^{-3}$ from around α=−8dB. On the other hand, the MLC-TPSR 2 is able to mitigate the IRI effect more than the Antipodal-TPSR, even when the IRI effect is increased from −10dB and guarantees the BER of $10^{-3}$ by α=−2dB. In particular, the performance of MLC-TPSR 3 is the same as the theoretical BER achieved at $E_{b}/N_{0}=-4\;\mathrm{dB}$ with $G_{p}=30$ by considering the IR-CCC of $|\beta_{\ell }|\approx 0$.

Next, we analyze the BER performance of the secondary relay. In the $\left|\beta_{\ell }\right|=1$ condition, since the secondary relay transmits the same signal of the primary relay, the BER performance is also the same as the primary relay in the SLC-TPSR, the Antipodal-TPSR, and the MLC-TPSR 1. On the other hand, the MLC-TPSR 2 is able to mitigate the IRI effect more than the Antipodal-TPSR even when the IRI is increased from −9dB and guarantees a BER of $10^{-3}$ by α=−4dB. In particular, the performance of MLC-TPSR 3 is the same as the theoretical BER achieved at $E_{b}/N_{0}=-4\;\mathrm{dB}$ with $G_{p}=30$ by considering the IR-CCC of $|\beta_{\ell }|\approx 0$. However, since the SR-CCC of the MLC for the secondary relay is larger than that of the primary relay, as shown in Table 1, the BER performance of the secondary relay is more deteriorated than the primary relay.

### 4.3. BER Performance with $E_{b}/N_{0}$

**Figure 5:**
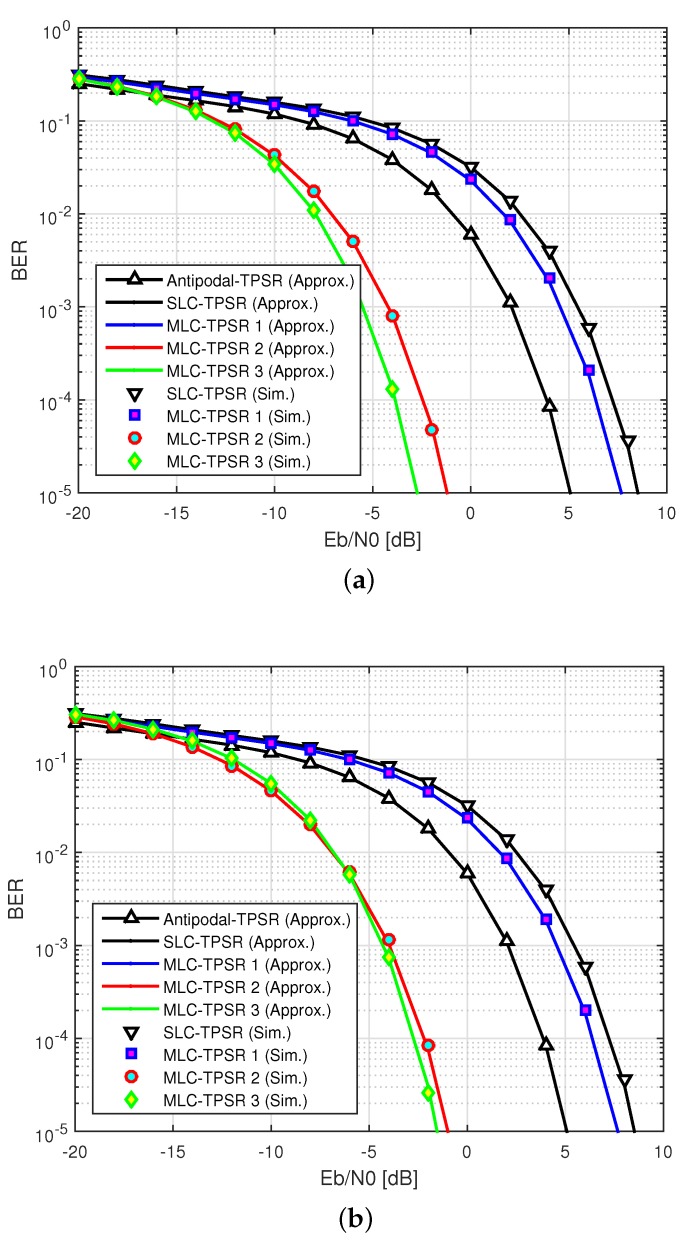
BER performance according to $E_{b}/N_{0}$$\left(\alpha =-3\;\mathrm{dB},\;G_{p}=30\right)$. (**a**) Primary relay; (**b**) secondary relay.

Figure 5 shows the BER performance according to $E_{b}/N_{0}$ with $G_{p}=30$ when α=−3dB. At first, we analyze the BER performance of the primary relay. From the results, since the Antipodal-TPSR has the antipodal property, the BER performance is improved over the SLC-TPSR and the MLC-TPSR 1, and is worse than the BER of $10^{-3}$ below $E_{b}/N_{0}=2\;\mathrm{dB}$. We observe that the BER performance of the MLC-TPSR 1 is degraded by about 3.5dB at a BER of $10^{-3}$ compared to the Antipodal-TPSR due to the SR-CCC. On the other hand, the BER performances of the MLC-TPSR 2 and the MLC-TPSR 3 are improved by about 6dB and 7.5dB, respectively, at a BER of $10^{-3}$, over the Antipodal-TPSR, mainly due to the IR-CCC. Next, we analyze the BER performance of the secondary relay. In the MLC-TPSR 3, due to the SR-CCC of the MLC, the BER performance of the secondary relay was worse than the primary relay. However, since the MLC-TPSR 2 and the MLC-TPSR 3 can mitigate the IRI effect, their BER performances are still better than the others. Consequently, we can conclude that the proposed scheme can significantly mitigate the IRI effect by considering the IR-CCC, even in high IRI environments.

## 5. Conclusions

In this paper, we analyzed the IRI effect in the CSS-based TPSR protocol. In order to mitigate the IRI effect, we considered the MLC signals. Then, we derived the IR-CCC according to the separating bandwidth and found the optimal separating bandwidth. From the simulation results, we verify the theoretical BER performance of the proposed scheme and conclude that the proposed scheme can mitigate the IRI effect even in high IRI environments, while performing full duplex transmission. Consequently, we have proposed a new paradigm to mitigate the IRI effect, and it is expected that the proposed scheme can help to design relay-based CSS systems for IoT communications.

## Figures and Tables

**Table 1:** Parameters of separating bandwidth pairs for each multiple linear chirp (MLC).

Type	Primary Relay		Secondary Relay		$\beta_{\ell }$
	$B_{\ell }\left[\mathrm{MHz}\right]$	$\rho_{\ell }$	$M_{\ell }\left[\mathrm{MHz}\right]$	$\rho_{\ell }$	
MLC-TPSR 1	2.6	−0.1121	2.6	−0.1121	1
MLC-TPSR 2	2.6	−0.1121	6.6	−0.0585	−0.273
MLC-TPSR 3	2.6	−0.1121	9.2	0.1513	0.007
